# Microstructure Evolution and Fatigue Properties of Ti Alloy Forged by 1500 t Forging Simulator

**DOI:** 10.3390/ma18071436

**Published:** 2025-03-24

**Authors:** Yoko Yamabe-Mitarai, Norie Motohashi, Shuji Kuroda, Prince Valentine Cobbinah

**Affiliations:** 1 Advanced Materials Science, Graduate School of Frontier Science, The University of Tokyo, 5-1-5, Kashiwanoha, Kashiwa 277-8561, Chiba, Japan; p.cobbinah22s@ams.k.u-tokyo.ac.jp; 2 Materials Fabrication and Analysis Platform, National Institute for Materials Science, 1-2-1, Sengen, Tsukuba 305-0047, Ibaraki, Japan; motohashi.norie@nims.go.jp (N.M.); kuroda.syuji@nims.go.jp (S.K.)

**Keywords:** TIMETAL834, forging, bimodal structure, spheroidization, globularization, fatigue, dwell fatigue

## Abstract

Microstructure control, especially the elimination of microtexture in Ti alloys such as Ti-6Al-4V and TIMETAL 834, is important to improve the fatigue life. In most research, small samples measuring 8–10 mm in diameter and 12–15 mm in height are utilized. However, the cooling rates of these small samples are always quite rapid, whereas the cooling rates of larger engine components, are relatively slow. Therefore, in this study, microstructural change involving different thermomechanical processing (TMP) was investigated using large TIMETAL 834 samples of 80 mm in diameter and 100 mm in height. The samples were forged at 940 and 1000 °C using a 1500 t forging simulator and heat treated at 900 and 1000 °C. Our goal is to attain a macroscopic understanding that connects the processing, microstructure, and fatigue life. The significant microstructure difference is that the deformed microstructure remains in the small sample due to rapid cooling, while the formation of a bimodal structure or an α phase globularization progressed in the large samples by diffusion during slow cooling. Improvement in the fatigue life was obtained by the 85% forging at 1000 °C. This is due to the refinement of the α grains and active slip in microtexture by alignment of the c-axis of α grains far from the tensile axis.

## 1. Introduction

Ti alloys are used in jet engine compressors due to their low density and good high-temperature mechanical properties [1]. Ti alloys are usually formed into a desired final shape and microstructure by thermomechanical processing (TMP), i.e., forging and heat treatment [2]. For high-temperature applications, a bimodal microstructure consisting of a 5–30% equiaxed α phase (HCP) and α/β (BCC) lamellar structure is desired to balance the creep and fatigue properties. A heterogeneous microstructure, where α grains are aligned with near crystallographic orientation, is often formed during TMP. This heterogeneous microstructure is referred to as a microtexture or macrozone. In a microtexture, grains with hard orientation align as the c-axis almost parallel to the tensile axis, and the slip system does not work due to the low Schmid factor [3]. A microtexture is surrounded by grains with soft orientations where the slip system becomes active.

The microtexture often acts as a crack initiation site and drastically reduces the fatigue life. Fatigue cracks have been observed to initiate in front of grains unfavorably oriented for prismatic <a> slip in the forged bar, unidirectionally rolled, and cross-rolled plate of Ti-6Al-4V [4]. The surface crack growth of forged Ti-6Al-4V has also been investigated by four-point bend fatigue testing [5]. If the crack planes are in good alignment with neighboring grains, a short crack can grow into a neighboring grain even with low Schmid factors during a fatigue test, and its growth in the microtexture can be non-deviated with minimal resistance. Additionally, microstructure, normal, and dwell fatigue lives have been investigated in alloys of varying Mo contents [3]. Crack initiation was often observed in the microtexture where a hard orientation region formed between soft regions. Of note, the microtexture decreased with the increase in the Mo contents from 2 to 6 mass %, resulting in longer fatigue lives. As a result, the life debit and the ratio of the normal and dwell fatigue lives decreased with the increase in Mo.

Recently, by directly observing crack initiation and propagation during fatigue tests, new mechanisms have been reported. For instance, in situ optical microscopy monitoring and electron backscattered diffractometry (EBSD) analysis of fatigue crack initiation and propagation in a bimodal forged Ti-6Al-4V revealed the occurrence of both transgranular and intergranular cracks [6]. Transgranular cracks are mainly initiated on basal or prismatic slip planes that have a high Schmid factor, while intergranular cracks form at tilt grain boundaries. Additionally, a significant observation noted was that the microtexture was not essential for crack initiation at 10 μm in size but was for crack propagation across the first few grain boundaries. Also, by quasi in situ observation with high resolution—digital image correlation (HR-DIC)—the effect of the primary α (α_p_) volume fraction and microtexture on fatigue crack initiation in TIMETAL 834 (Ti-5.8Al-4Sn-3.5Zr-0.7Nb-0.5Mo-0.35Si-0.06C) was investigated [7]. It was found that intergranular crack initiation often preceded transgranular crack initiation, and an increase in the volume fraction and size of the primary α phase decreased the crack initiation life. Moreover, the authors asserted that the (0001) twist grain boundary could be a crack initiation site of intergranular cracking by combining the preference neighboring α grains pair for basal <a> slip.

Furthermore, extensive investigations into large forged near-α Ti billets have been performed to understand the formation mechanisms of the microtexture [8,9,10,11]. Microstructural characterization revealed two different texture areas, i.e., microtexture and randomly oriented areas developed in a forged bimodal Ti60 (Ti-5.7Al-3.7Sn-3.5Zr-0.4Mo-0.4Nb-1.0Ta-0.4Si-0.05C) billet [8]. The observed microtexture corresponded to remnants of the lamellar structure, while the randomly oriented area corresponded to the equiaxed α grains (α_p_) formed by severe deformation during forging. Along similar lines, in forged Ti6242S, the appearance of a flow localization band resulted in microtexture evolution [9]. This flow localization band included much higher geometric dislocation densities (GNDs) and a favorable prismatic <a> slip system causing the formation of the microtexture. In addition, in a detailed investigation of the longitudinal section of a TIMETAL 834 billet, the microtexture formed along the elongation direction [10]. The equiaxed α grains (α_p_) were oriented around a single texture component with about 20° spread in the microtexture. On the other hand, the α phase in the lamellar structure (α_s_) exhibited a more significant number of textures than α_p_ grains. It was concluded that the microtexture formation was due to the overlapping of the quasi single-crystal α_p_ orientation with the main α_s_ texture component comprising 70% of the material. Microstructural variations in bimodal forged TIMETAL 834 disks have also been investigated [11]. Three different areas involving the dead zone, which was almost unaffected by the forging process, the central part of the dies that was severely deformed by compression, and the intermediately deformed region close to the rim were observed. The microtexture was strongly observed along the forged direction in the dead zone. In the medium-deformed region, the microtexture remained elongated along the forged direction. Conversely, the microtexture weakened in the severely deformed region, indicating severe deformation coincided with the elongation direction of the billet, which effectively eliminated the microtexture. Furthermore, the mentioned study demonstrated that the microtexture would favorably form when the α_p_ and the prior- β grains share a Burgers orientation relationship (BOR), due to the preferential growth of α_s_ in the prior-β grains. In addition, a slow cooling rate promoted microtexture development because only a few α_s_ variants formed during slow cooling.

One drawback of the published studies involving large forged billets is the deliberate generalization of the processing conditions aimed at protecting an industry’s know-how. To circumvent this challenge, numerous studies use small compression specimens of approximately 8–10 mm in diameter and 12–15 mm in height, and by changing the condition of the compression test systematically, the microstructure change can be thoroughly investigated [12,13,14,15,16]. An example includes the effect of the strain rate on TIMETAL 834 microstructure, forged at 1000 °C in the α + β two-phase regions investigated by Kodli et al. [12]. The microtexture formed in the received sample and varied significantly with the strain rate. The different slip systems, depending on strain rate, resulted in various microtextures. Another example involves the TIMETAL 834 microstructural comparison between a compressed sample at 1000 °C and a heat-treated sample at 1000 °C [13]. Three main factors that form a single orientation of α_p_, microtexture, were suggested: (1) the initial colonies deform in the same way; (2) the deformation of HCP leads to sharp texture; and (3) the α_p_ globularization produces a few new orientations. Two main factors that develop the strong texture of the α_s_ were also suggested: (1) neighboring β and α_p_ grains have BOR, and (2) the colonies with the lamellar structure are preferentially selected to share the same orientations as the surrounding α_p_ during transformation. These mechanisms correspond with those suggested in Ref. [11]. The strain rate and processing temperature effects of Ti-65 alloy (Ti-5.8Al-4.0Sn-3.5Zr-1.0Ta-0.3Nb-0.5Mo-0.5Si-0.8 W) have also been investigated [14]. It was found that the texture strength of α_p_ and α_s_ becomes stronger with an increase in temperature from 950 °C, because the volume fraction of the α_p_ decreases, and variant selection occurs at 1010 °C. With an increase in the strain rate, the texture strength of the α_p_ phase gradually decreases, because the increase in the lattice rotation decreases the texture evolution. On the contrary, in α_s_, the texture intensity increased with an increased strain rate, because the dynamic globularization of α_s_ decreased with an increased strain rate. Similar results were obtained in Ti-65 in Ref. [15]. The effect of the forging ratio on the microstructure of Ti6242S (Ti-6Al-2Sn-4Zr-2Mo-0.08Si) has also been investigated at 970 °C [16]. The separation of the α_p_ grains by dynamic recrystallization (DRX) or dynamic recovery (DRC) caused refining grains with the increase in the deformation strain. DRX could soften the texture intensity. In addition, the microtexture becomes weak above a 50% forging ratio, because the DRX increased with increasing forging ratio.

As highlighted above, appreciable progress has been made in understanding the formation mechanisms behind microtexture evolution and the influence of processing conditions on the microstructure using small samples with 8–10 diameter and 10–15 mm height. However, the cooling rates of small samples are always rapid, while the cooling rate of large engine parts like turbine disks is relatively slow in comparison [17]. Since the microstructure of Ti alloys strongly depends on the cooling rate, an investigation of the microstructure using large compression test specimens is warranted. Therefore, in this study, microstructural change involving different thermomechanical treatments was investigated using large samples of 80 mm in diameter and 100 mm in height. The fatigue life was also investigated. Our goal is to attain a macroscopic understanding by connecting the processing, microstructure, and fatigue life.

## 2. Materials and Methods

The TIMETAL 834 billet of 300 mm in diameter and 300 mm in height was purchased from TIMET. The alloy composition is Ti-5.8Al-4Sn-3.5Zr-0.5Mo-0.7Nb-0.35Si-0.06C wt%. Compression specimens of dimensions 80 mm in diameter and 100 mm in height and 8 mm in diameter and 12 mm in height were sectioned from the billet. The microstructure of the as-received billet was observed using specimens of 8 mm in diameter and 12 mm in height obtained in the area of 60 mm off the center, i.e., 1/2 of the radius. The specimens of 80 mm in diameter and 100 mm in height were forged under various conditions using a 1500 t forging press, followed by air cooling. The forged samples were then sectioned into two semicircular plates, and each semicircular plate was heat treated at 900 and 1000 °C for 2 h, respectively, followed by air cooling. The thermomechanical processing is shown in Table 1. To compare with the microstructures, the small specimens of 8 mm in diameter and 12 mm in height were compressed to 75% at 940 and 1000 °C using a 25 t forging press (THERMECMASTER, Fuji Electronic Industrial Co., Ltd., Tsurugashima, Japan). The strain rates used included 0.005, 0.05, and 0.5/s. The samples were quickly cooled using hydrogen gas. The heat treatment was performed for the forged samples at 900 and 1000 °C for 2 h, followed by air cooling.

**Figure 1:**
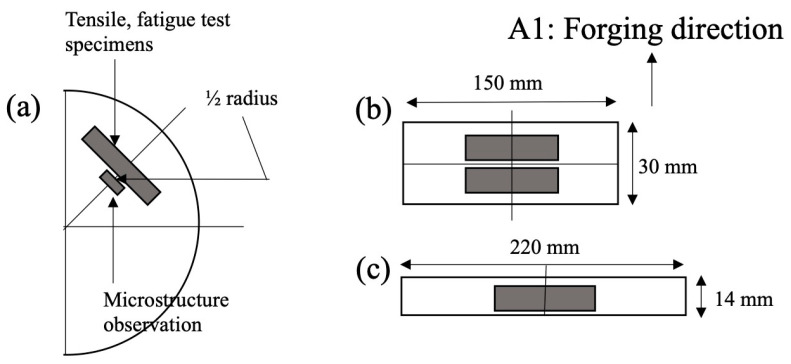
Specimen pick-up region from the forged sample. (**a**) From the upper direction of the semicircle plate, (**b**) cross section of 70% forged samples, and (**c**) cross section of 85% forged samples.

The samples for microstructure observation and tensile and fatigue tests were sectioned from the heat-treated samples. The sample pick-up region is shown in Figure 1.

For microstructure observation, the sample was first embedded in a resin and then polished using sandpaper, diamond suspensions from 9, 6, and 3 μm, and finally, SiO_2_ solution. The microstructure observation was performed using the scanning electron microscope (SEM, 7200F, JOEL, Akishima, Japan) with an accelerating voltage of 20 kV.

The tensile and fatigue specimens of 6 mm in diameter and 30 mm in measurement were taken from the forged semicircular plate. The tensile test was performed using 10 MPa/s up to the yield strength and 20% /mins up to fracture at room temperature. Normal and dwell fatigue tests were also carried out. The conditions used for the normal fatigue test included 0.9σ of 0.2% proof stress (σ), the ratio of minimum and maximum stress, R = 0.1, and 5 Hz. In addition, the dwell fatigue was carried out with the same condition as the normal fatigue, but the holding time of the maximum stress was 120 s, and the time increase from 0.09 σ to 0.9 σ was 1 s.

## 3. Results

### 3.1. Microstructure of As-Received Billet and Forged Samples by 1500 t Forging Press

**Figure 2:**
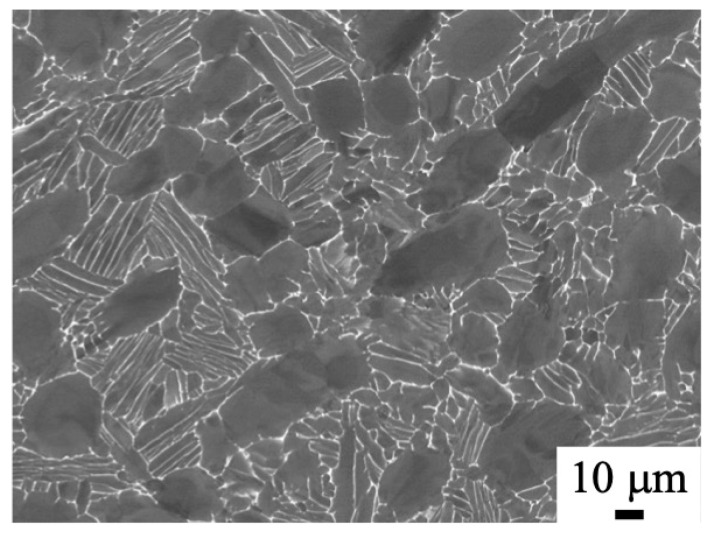
SEM backscattered electron image of the normal plane of the as-received billet.

The microstructure of the as-received billet is shown in Figure 2. A typical bimodal structure consisting of an equiaxed α phase and α/β lamellar structure was observed.

**Figure 3:**
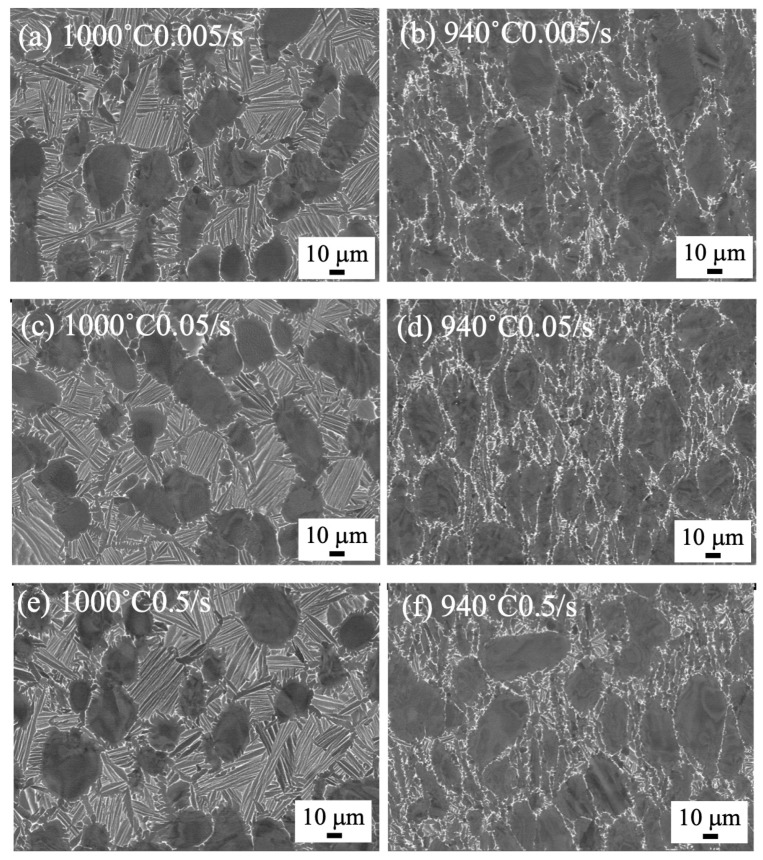
SEM backscattered electron images of forged to 70% at 1000 °C (**a**,**c**,**e**) and 940 °C (**b**,**d**,**f**) with a strain rate of 0.005/s (**a**,**b**), 0.05/s (**c**,**d**), and 0.5/s (**e**,**f**), using 1500 t forging press.

**Figure 4:**
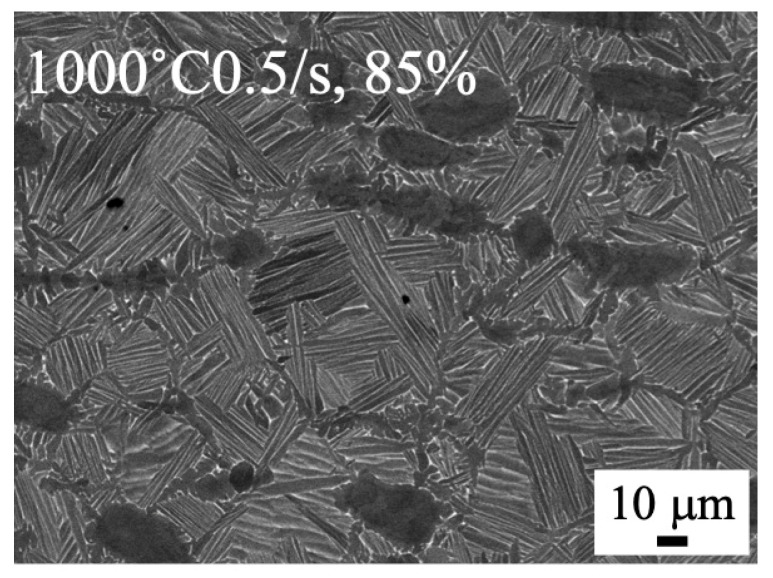
SEM backscattered electron images of forged to 85% at 1000 °C with a strain rate of 0.5/s, using 1500 t forging press.

The microstructures of the forged samples of 80 mm in diameter and 100 mm in height to 70% are shown in Figure 3. The microstructure was clearly different at the forging temperature but showed no difference in strain rate. The bimodal structure remained after forging at 1000 °C, but the α/β lamellar structure was broken after forging at 940 °C. The microstructure of the forged samples to 85% is shown in Figure 4. The bimodal structure remained as well as in the sample with the 70% compression ratio.

### 3.2. Microstructure of Forged Samples by 25 t Forging Press

**Figure 5:**
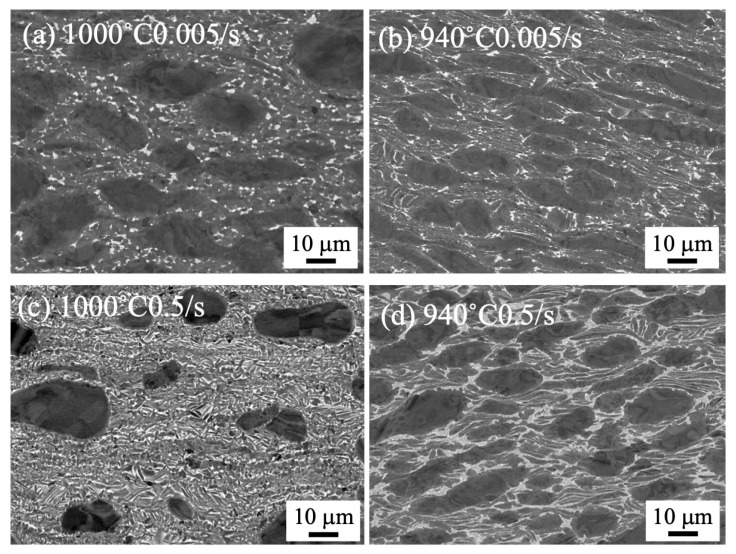
SEM backscattered electron images of forged to 75% at 1000 °C (**a**,**c**) and 940 °C (**b**,**d**) with a strain rate of 0.005/s (**a**,**b**) and 0.5/s (**c**,**d**), using 25 t forging press.

To compare with the microstructures of small samples of 8 mm in diameter and 12 mm in height, the microstructures of samples forged by the 25 t forging press are shown in Figure 5. As noticed, the microstructure drastically changed. A severely deformed microstructure was observed after forging at 900 °C, regardless of the strain rate. By forging at 1000 °C with a strain rate of 0.005/s, the α/β lamellar structure was divided, and the β phase with bright contrast started globularizing (Figure 5a). By forging at 1000 °C with a strain rate of 0.5/s, the dividing of α/β lamellar structure started to occur, but the α/β lamellar structure remained (Figure 5c).

### 3.3. Microstructure of Heat-Treated Samples Forged by 1500 t Forging Press

**Figure 6:**
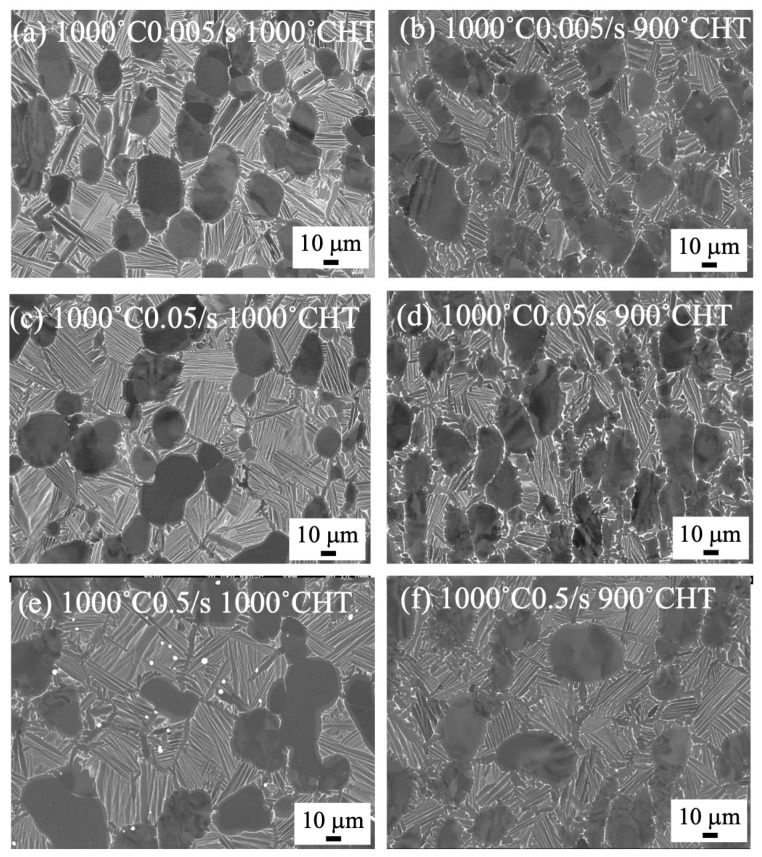
SEM backscattered electron images of forged to 70% at 1000 °C and heat treated at 1000 °C (**a**,**c**,**e**) and 900 °C (**b**,**d**,**f**) with a strain rate of 0.05/s (**c**,**d**) and 0.5/s (**e**,**f**), using 1500 t forging press.

**Figure 7:**
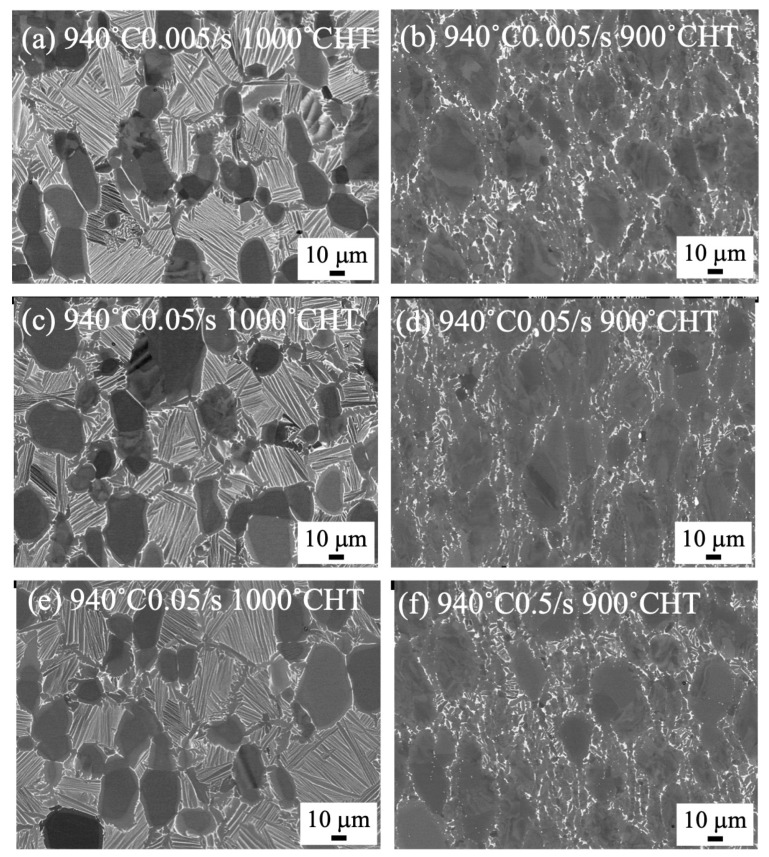
SEM backscattered electron images of forged to 70% at 940 °C and heat treated at 1000 °C (**a**,**c**,**e**) and 900 °C (**b**,**d**,**f**) with a strain rate of 0.005/s (**a**,**b**), 0.05/s (**c**,**d**), and 0.5/s (**e**,**f**), using 1500 t forging press.

**Figure 8:**
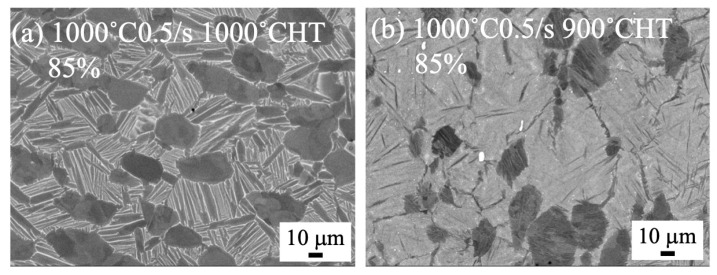
SEM backscattered electron images of forged to 85% at 1000 °C and heat treated at (**a**) 1000 °C and (**b**) 900 °C with a strain rate of 0.5/s, using 1500 t forging press.

The microstructure of heat-treated samples forged at 1000 °C and 940 °C to 70% by a 1500 t forging press are shown in Figure 6 and Figure 7, respectively. Regardless of the heat treatment temperatures at 900 and 1000 °C, the bimodal structures were formed for the forged samples at 1000 °C (Figure 6). On the other hand, the microstructure of the heat-treated samples of the samples forged at 940 °C drastically changed. The bimodal structure was formed after heat treatment at 1000 °C, while separation of the α/β lamellar structure was observed after heat treatment at 900 °C (Figure 7). In Figure 8, the microstructure of the heat-treated samples forged at 1000 °C to 85% are shown. The bimodal structure formed in the heat-treated sample at 1000 °C after forging with 0.5/s to 85%. By heat treating at 900 °C, the stable β phase at 900 °C transformed into the α phase during cooling, and then a plate-like α phase was observed in the β phase.

**Figure 9:**
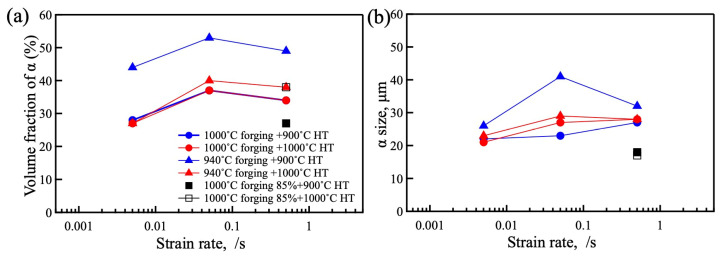
Comparison of the α phase (**a**) volume fraction and (**b**) size after the different processing conditions.

The volume fraction and size of the α phase in the heat-treated samples were measured using Image J (version 1.53k) and are plotted in Figure 9a and Figure 9b, respectively. The α volume fractions of samples forged at 1000 °C were almost the same after heat treatment at 900 and 1000 °C. The α volume fraction of the sample forged at 940 °C and heat treated at 1000 °C was almost the same as the sample forged at 1000 °C, while the α volume fraction of the sample forged at 940 °C and heat treated at 900 °C was higher than the other conditions. For the sample forged at 1000 °C to 85%, the sample heat treated at 900 °C indicated a lower α volume fraction compared with the other conditions, while the α volume fraction of the sample heat treated at 1000 °C had a similar value to the other forged sample heat treated at 1000 °C. The size of the α phase measured between 20 and 40 μm with no large difference observed as shown in Figure 9b.

### 3.4. Mechanical Properties

**Figure 10:**
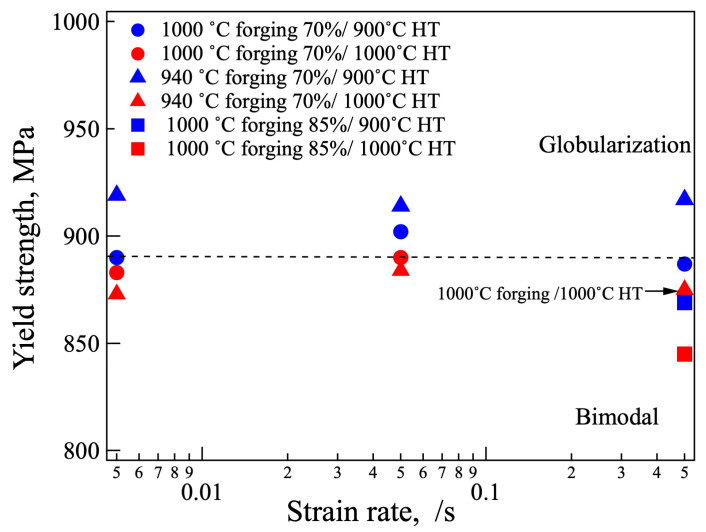
The 0.2% proof stress of forged and heat-treated samples.

The 0.2% proof stresses are plotted in Figure 10. The 0.2% proof stresses were between 850 and 920 MPa. The samples with a globular α phase formed after heat treatment at 900 °C had a relatively higher strength among the samples forged to 70%. The 0.2% proof stress of the F1000-C-1000H overlapped with F940-C-1000H. Furthermore, the 0.2% proof stress was insensitive to the strain rate. The sample forged to 85% was clearly lower than those forged to 70%.

The fatigue test condition was determined from the 0.2% proof stress. The maximum and minimum applied stresses are summarized in Table 2. The fatigue tests failed for samples forged at 940 °C to 70% with a strain rate of 0.005/s.

**Figure 11:**
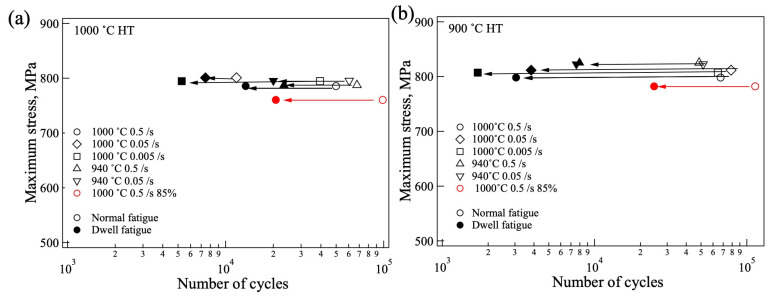
Fatigue life of the sample heat treated at (**a**) 1000 °C and (**b**) 900 °C.

The lives of normal and dwell fatigue are shown in Figure 11. For the samples heat treated at 1000 °C (Figure 11a), the normal fatigue life was almost of the same value, although the normal fatigue life of the sample forged at 1000 °C and strain rate of 0.05/s (1000F-B-1000H) indicated a lower, almost one order, difference for some reason (maybe, the fatigue test failed). The dwell fatigue lives were all lower compared with those with normal fatigue. For the sample heat treated at 900 °C (Figure 11b), a similar behavior was observed. The normal fatigue lives were almost the same as those of the sample heat treated at 1000 °C, but the dwell fatigue lives drastically decreased compared with those heat treated at 1000 °C. The sample forged at 1000 °C to 85% exhibited longer fatigue lives for both normal and dwell fatigues.

**Figure 12:**
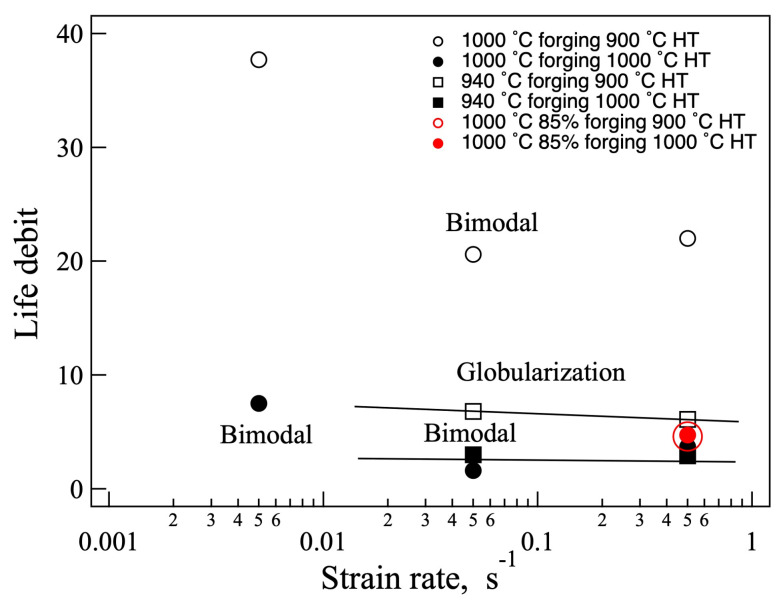
Life debit of the forged and heat-treated samples.

It is important to know the difference between the normal and dwell fatigue lives. Hence, the ratio of the normal fatigue life and the dwell fatigue life, the life debit, was calculated and is shown in Figure 12. The samples heat treated at 1000 °C exhibited a relatively small life debit, while the samples heat treated at 900 °C showed a relatively high life debit. The life debit of the samples heat treated at 900 °C after 1000 °C forging was significantly high through all the strain rate ranges. No significant difference was observed between the compressed ratio of the 70 and 85% forged and heat treated at 1000 °C.

## 4. Discussion

### 4.1. Microstructure Formation of Forged Samples

**Figure 13:**
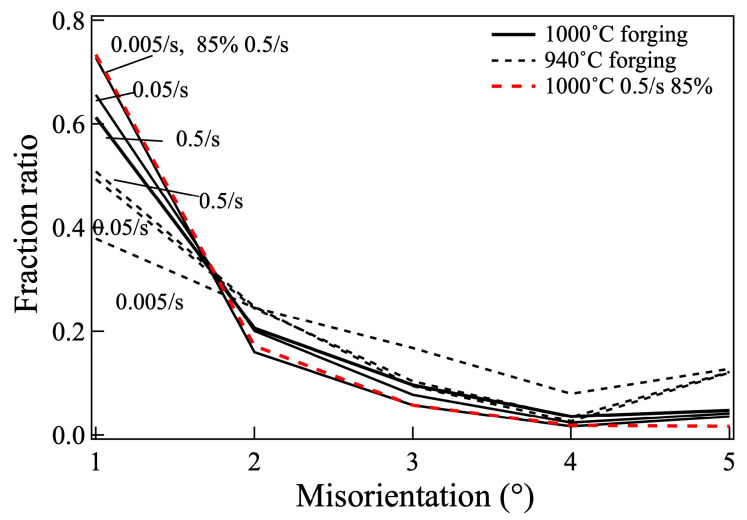
Misorientation distribution in the forged samples.

The forged microstructure depended on the forging temperature but not on the strain rate or forging ratio, as shown in Figure 3 and Figure 4. During forging at 900 °C, the α/β lamellar structure was broken severely, and globularization of the α phase occurred (Figure 3). To understand the microstructure formation, Kernel Average Misorientation (KAM) maps were drawn using TSL analysis software, ver. 8. The fraction ratio of the misorientation obtained from the KAM maps is plotted in Figure 13. It is very clear that the forged samples at 940 °C indicate a relatively high fraction ratio for high misorientation and a low fraction ratio for low misorientation compared with the forged samples at 1000 °C. Furthermore, when the strain rate was slow, 0.005/s at 940 °C, the fraction ratio of the high misorientation became higher than in other samples. On the contrary, at 1000 °C, the fraction ratio for low misorientation was higher, and the ratio for high misorientation was lower at a slower strain rate. For the sample forged at 1000 °C to 85%, the distribution of misorientation was almost the same as those at 1000 °C with 0.005/s. This indicates that the induced strain during forging was higher at 940 °C. The high misorientation, i.e., induced strain, destroyed the α/β lamellar structure and progressed the globularization of the α grains.

**Figure 14:**
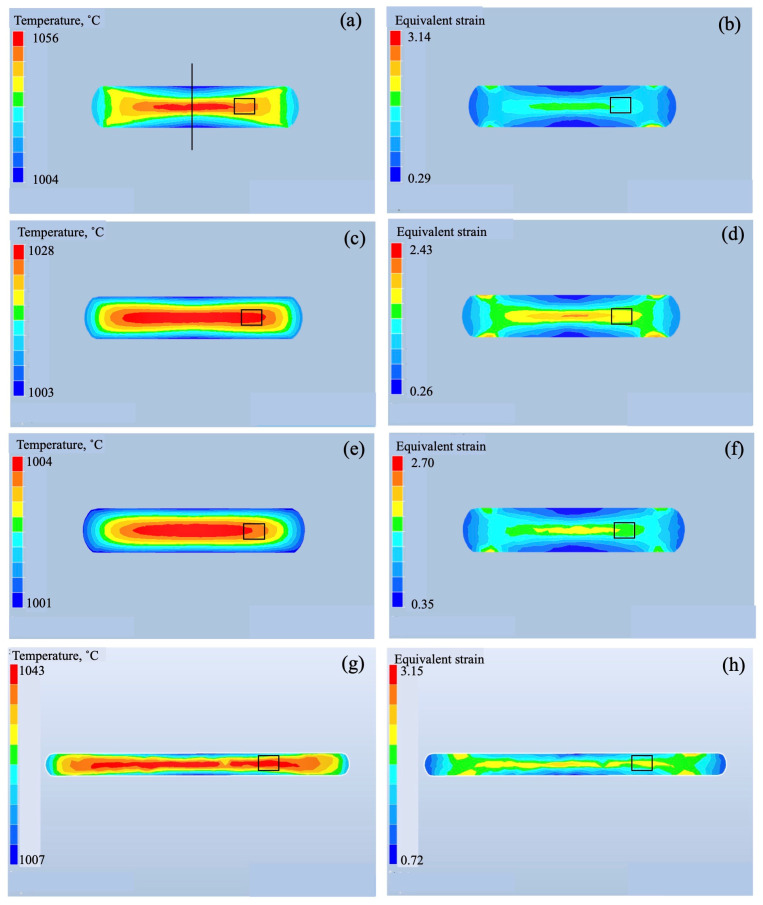
Temperature (**a**,**c**,**e**,**g**) and equivalent strain (**b**,**d**,**f**,**h**) distribution of the samples forged at 1000 °C. The strain rates used were 0.5/s (**a**,**b**), 0.05/s (**c**,**d**), and 0.005/s (**e**,**f**). The forging ratio is 70% for (**a**–**f**) and 85% for (**g**,**h**). The black line indicates center of sample.

**Figure 15:**
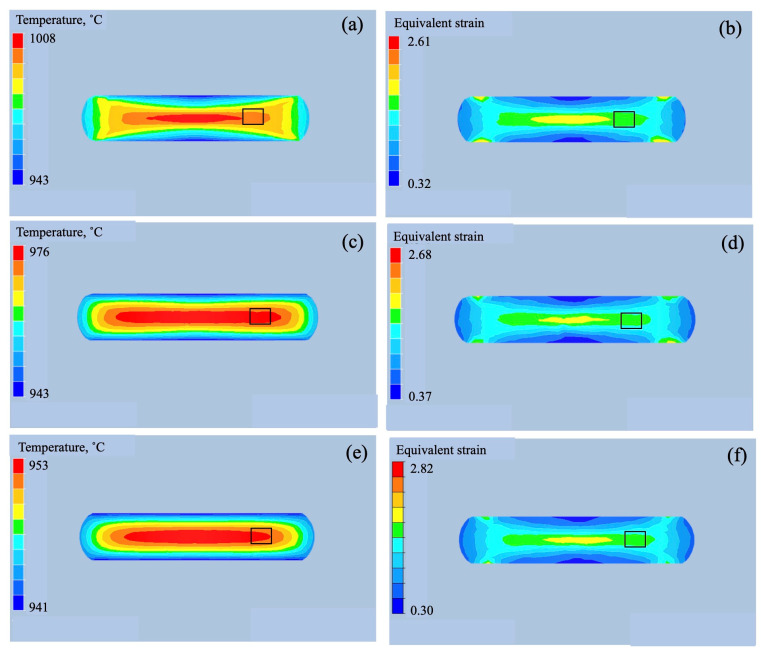
Temperature (**a**,**c**,**e**) and equivalent strain (**b**,**d**,**f**) distribution of the samples forged at 940 °C to 70%. The strain rate is 0.5/s (**a**,**b**), 0.05/s (**c**,**d**), and 0.005/s (**e**,**f**). The black line indicates center of sample.

**Figure 16:**
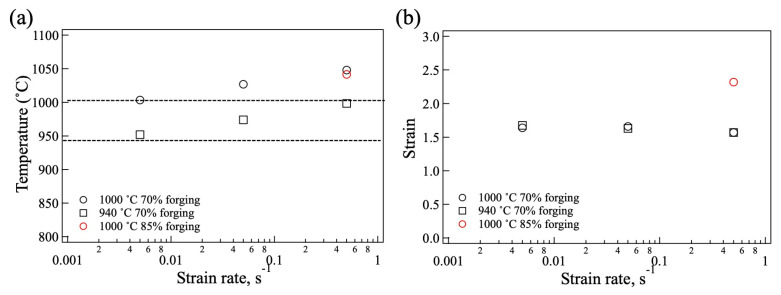
(**a**) Temperature and (**b**) equivalent strain of the forged samples in the area of 1/2 radius.

To understand the macroscopic strain change during forging, the temperature and strain distribution in the samples were calculated using finite element method (FEM) simulation (FORGE NxT 3.2, Transvalor, Antipolis, France). The Hansel Spittel approximate expression was used as the material model. The specific heat: 0.525 J/gK, density: 4.55 g/cm^3^, and thermal conductivity: 7.06 W/mK were used for calculation. The results at 1000 °C and 940 °C are shown in Figure 14 and Figure 15, respectively. It is evident that processing heat was generated during forging, and the area of the processing heat becomes larger with a decrease in the strain rate at both processing temperatures. By forging to 85%, the wide area generated processing heat (Figure 14g). On the other hand, the equivalent strain did not change under the processing conditions. To explicitly show this change quantitatively, the temperature and equivalent strain in the area outlined with the rectangle, 1/2 of radius, are plotted in Figure 16. The dotted lines represent the forging temperature. As shown in Figure 16a, the processing heat increased with the increase in the strain rate. At a strain rate of 0.5/s, the temperature increased by 58 °C and 48 °C during forging at 940 °C and 1000 °C, respectively, while at a strain rate of 0.005/s, the temperature did not change significantly. The process heat of the 85% forged sample was almost the same as that of the 70% forged samples. The equivalent strain showed no change in strain rate, as depicted in Figure 16b, but changed per the forging ratio. The equivalent strain of the 85% forged sample was higher than that of the 70% forged samples.

Compared with the observed misorientation distribution in Figure 13, the high strain rate generates large process heat in the sample forged at 940 °C, and then strain relaxation occurs. As a result, the strain distribution at the high strain rate at 940 °C shifted to a low strain distribution. For 1000 °C forging, although process heat was generated, the misorientation distributions above 2% were similar. This indicates that 1000 °C was high enough for strain relaxation, and the process heat did not affect the equivalent strain.

It is considered that the α/β lamellar structure was destroyed by microstructure distortion during forging at 940 °C, but the segmented α phase spheroidized at 940 °C. The microstructure observation in Figure 3b,d,f did not show dependence on the strain rate, suggesting the process heat did not affect the microstructure formation. The bimodal structure remained in the 1000 °C forging irrespective of the strain rate and the amount of process heat. It is considered that microstructure distortion occurred during forging, but dynamic recovery followed soon after, and the bimodal structure formed during slow cooling from the equiaxed α + β structures.

Comparing the forged microstructure of the small and large samples in Figure 3 and Figure 5, the microstructure distortion was obvious in the small samples forged by the 25 t forging press in Figure 5. This indicates the deformed microstructure remained, due to the fast cooling rate in the small sample, while the large sample forged by the 1500 t forging press showed no distorted microstructure, suggesting the microstructure changed during slow cooling. In all, the comparison underscores the importance of the sample size in understanding the microstructure change during thermomechanical processing.

### 4.2. Microstructure Formation of Heat-Treated Samples

By heat treating at 1000 °C, the bimodal structure formed regardless of the forging temperature (Figure 6a,c,e and Figure 7a,c,e). Conversely, by heat treating at 900 °C, the bimodal structure formed in the 1000 °C forged samples (Figure 6b,d,f), but the globularization of the α phase was observed in the samples forged at 940 °C (Figure 7b,d,f). The strain rate showed no influence on the heat-treated microstructure. This indicates that recrystallization of the α and β phases occurred at 1000 °C in the samples forged at 940 and 1000 °C. In addition, an α/β lamellar structure formed during slow cooling in the β phases, resulting in the formation of the bimodal structure. The globularization of the α phase in the samples heat treated at 900 °C indicated that recrystallization was not enough at 900 °C.

**Figure 17:**
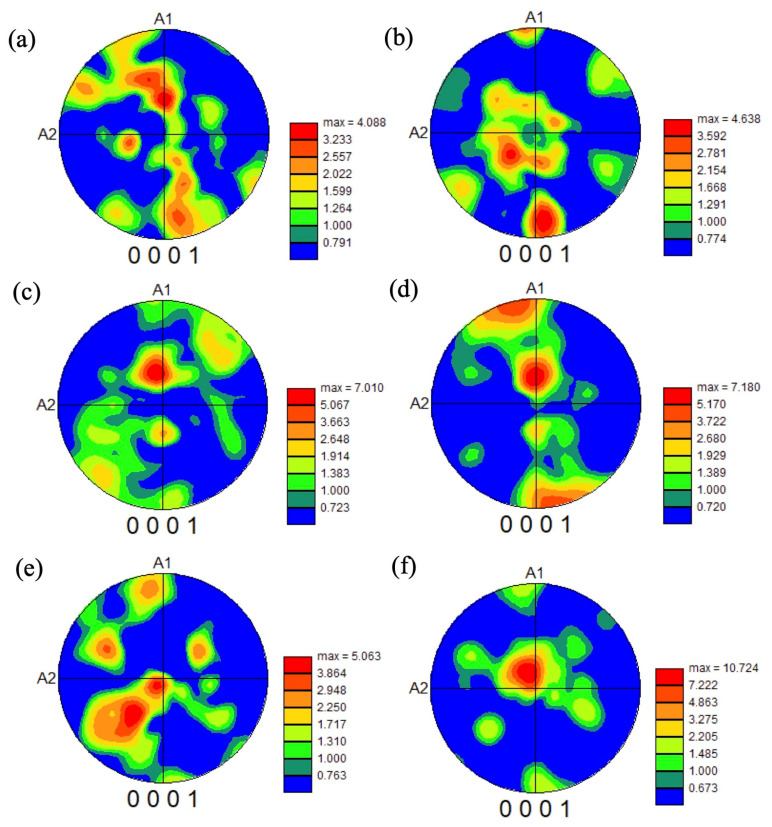
Pole figures of the basal plane in the samples forged at 1000 °C to 70% with strain rates of 0.005/s (**a**,**b**), 0.05/s (**c**,**d**), and 0.5/s (**e**,**f**), heat treated at 1000 °C (**a**,**c**,**e**) and 900 °C (**b**,**d**,**f**).

**Figure 18:**
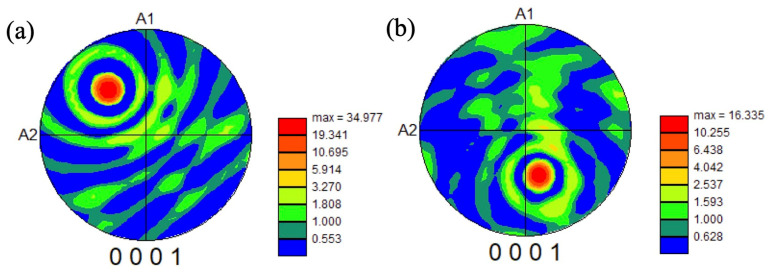
Pole figures of the basal plane in the samples forged at 1000 °C to 85% with a strain rate of 0.5/s, heat treated at (**a**) 1000 °C and (**b**) 900 °C.

**Figure 19:**
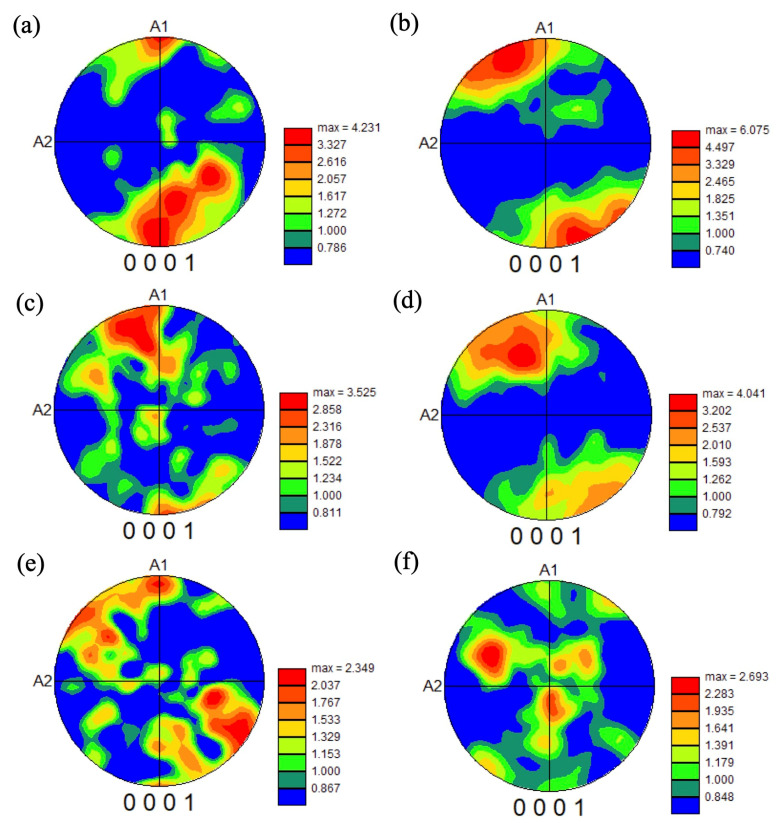
Pole figures of the basal plane in the samples forged at 940 °C to 70% with strain rates of 0.005/s (**a**,**b**), 0.05/s (**c**,**d**), and 0.5/s (**e**,**f**), heat treated at 1000 °C (**a**,**c**,**e**) and 900 °C (**b**,**d**,**f**).

To investigate the texture in the heat-treated samples, the pole figures were drawn for heat-treated samples after forging at 1000 °C, as shown in Figure 17 and Figure 18, and forging at 940 °C, as shown in Figure 19.

For the samples forged at 1000 °C, the texture of the (0001) basal plane was observed in all the heat-treated samples. Although some basal planes aligned to A1 (forged direction), in most cases, the basal planes aligned far from the A1 direction, indicating the occurrence of an active slip on the basal plane and the decrease in crack initiation near the microtexture. The texture intensity was relatively higher in the sample heat treated at 900 °C and higher with a high strain rate in the samples heat treated at 900 °C. Compared with the forging ratio of 70 and 85% (Figure 17a,b and Figure 18), the texture intensity was higher in the 85% forged sample. Generally, a high texture intensity indicates a high-volume fraction of microtexture, and the crack initiation site is increased. However, in the samples forged at 1000 °C, the basal plane was not aligned close to the normal direction of the tensile axis, and the microtexture does not become a crack initiation site. Then, the existence of the microtexture might not affect the fatigue life.

For the samples forged at 940 °C, similar to the sample forged at 1000 °C, some basal planes aligned in the A1 direction, but most of the basal planes aligned far from the A1 direction. Furthermore, the texture intensity in the 940 °C forged samples was smaller than that in the samples forged at 1000 °C, indicating that a random structure formed in the 940 °C forged samples. These trends are similar to those reported by Ref. [18]. Under large pre-deformation, the BOR between the α_p_ and neighboring β grains was destroyed, due to the rotation of the α_p_ grains. Hence, the degree of variant selection of α_s_ colonies decreased significantly, reducing the texture development. In our results, the globularization of the α phase in the samples heat treated at 900 °C indicates the BOR was destroyed during forging at 940 °C. Even so, by heat treating at 1000 °C, the microstructure recovered to equiaxed α and β grains. The texture intensity of the sample forged at 940 °C and heat treated at 1000 °C was almost the same value as the sample forged at 1000 °C (Figure 17a,c,e).

### 4.3. Effect of Microstructure on Fatigue Life

The influence of the processing conditions and resulting microstructures on fatigue life can be summarized as follows: (1) The normal fatigue lives were almost the same for different microstructures, such as bimodal and globularized α grains. (2) The dwell fatigue lives of the samples heat treated at 900 °C were relatively lower, regardless of bimodal or globularized α grains. (3) The normal and dwell fatigue lives of the 85% forged sample were relatively longer than the other processing conditions, even after heat treatment at 900 °C. The relationship between the volume fraction and size of the α_p_ phase and crack initiation was suggested in Ref. [7]. Increasing the α_p_ volume fraction and size caused an increase in intergranular cracks at the (0001) twist grain boundary. In our study, the volume fraction and size of the α_p_ phase were relatively higher in the sample heat treated at 900 °C than in the samples heat treated at 1000 °C, because the α phase is more stable at 900 °C from the point of phase equilibrium. This suggests the crack initiation site increased in the sample heat treated at 900 °C. Most studies usually focus on crack initiation and initial propagation, while fatigue life is rarely discussed. Based on such interest, it becomes difficult to discuss fatigue life directly from crack initiation. However, if the crack initiation site increases, it is expected that propagation also occurs from these sites and that the fatigue life will become shorter. Microtexture is not a necessary condition for crack initiation, but it is a necessary condition for their propagation across the first few grain boundaries [6]. The texture intensity after heat treatment at 900 °C is slightly higher, especially for forging at 940 °C, suggesting crack propagation becomes easier in the microtexture, causing a low dwell fatigue life in the samples heat treated at 900 °C.

Both the normal and dwell fatigue lives of the 85% forged samples were longer than those of the 70% forged samples. The large difference between the microstructure of the 85% forged samples and 70% forged samples is the smaller α grain size in the 85% forged samples due to the large induced strain during forging, as shown in Figure 16b. High strain enhanced dynamic recrystallization and formed fine grains. The decrease in the α grain size causes decreasing slip band spacing [3]. A smaller spacing of the slip bands meant a more homogeneous distribution of the dislocations, weakening the stress concentrated at the grain boundary. This contributes to reducing the propensity for crack formation. Thus, fine grains and random grain orientation improve fatigue life due to inhibition of crack propagation [3].

The optimized processing parameters are suggested to eliminate the microtexture [18]. With increased deformation, superplastic deformation through grain boundary sliding was activated in the refined grain sample. This superplastic deformation inhibits the inhomogeneous dislocation slip and randomizes the orientation of grains, resulting in the elimination of the microtexture. Large deformation that destroyed the BORs between α_p_ and their neighboring β grains led to a significant decrease in the degree of the variant selection of α_s_ colonies, thus reducing the development of texture [19]. From these mentioned findings and our results, we can assert that an increase in the forging ratio is effective in producing a fine-grain microstructure and less microtexture, resulting in an improvement in the fatigue life.

## 5. Conclusions

TIMETAL 834 samples measuring 80 mm in diameter and 100 mm in height were deformed to 70 and 85% at 940 and 1000 °C with strain rates of 0.005, 0.05, and 0.5/s for the duration of deformation of 12 s to 180 s. The thermomechanical conditions, microstructures, and fatigue life (normal and dwell) were investigated. A bimodal structure was observed in the samples forged at 1000 °C to 70% and 85% regardless of the strain rate, while α globularization was observed in the samples forged at 940 °C. The microstructure of the samples forged at 1000 °C did not change after heat treatments, both at 900 and 1000 °C. Conversely, for the microstructure of the samples forged at 940 °C, a bimodal structure formed by heat treatment at 1000 °C, but α globularization occurred by heat treatment at 900 °C. The microtexture was observed, but the orientation of the basal plane was not normal to the forging axis, resulting in an active slip system. The life debit between the normal and dwell fatigues was lower than eight. Longer normal and dwell fatigue lives in the 85% forged sample were caused by the fineness of the microstructure, due to the large strain induced during forging.

## Figures and Tables

**Table 1:** The thermomechanical process conditions for 1500 t forging press.

Sample Name	Forging Temperature, °C	Strain Rate, s^−1^	Compressed Ratio	Heat Treatment, °C
F940-A-H900	940	0.005	70	900
F940-A-H1000	940	0.005	70	1000
F940-B-H900	940	0.05	70	900
F940-B-H1000	940	0.05	70	1000
F940-C-H900	940	0.5	70	900
F940-C-H1000	940	0.5	70	1000
F1000-A-H900	1000	0.005	70	900
F1000-A-H1000	1000	0.005	70	1000
F1000-B-H900	1000	0.05	70	900
F1000-B-H1000	1000	0.05	70	1000
F1000-C-H900	1000	0.5	70	900
F1000-C-H1000	1000	0.5	70	1000
F1000-D-H900	1000	0.5	85	900
F1000-D-H1000	1000	0.5	85	1000

**Table 2:** The applied stresses for fatigue test.

Sample Name	Forging Temperature, °C	Strain Rate, s^−1^	Heat Treatment Temperature, °C	Maximum Stress, MPa	Maximum Stress, MPa
F940-B-H900	940	0.05	900	822.6	82.3
F940-B-H1000	940	0.05	1000	795.6	79.6
F940-C-H900	940	0.5	900	825.3	83.5
F940-C-H1000	940	0.5	1000	787.5	78.8
F1000-A-H900	1000	0.005	900	807.3	80.7
F1000-A-H1000	1000	0.005	1000	794.7	79.5
F1000-B-H900	1000	0.05	900	811.8	81.2
F1000-B-H1000	1000	0.05	1000	801.0	80.1
F1000-C-H900	1000	0.5	900	798.3	79.8
F1000-C-H1000	1000	0.5	1000	785.7	78.6
F1000-D-H900	1000	0.5	900	782.1	78.2
F1000-D-H1000	1000	0.5	1000	760.5	76.1

## Data Availability

The original contributions presented in this study are included in the article. Further inquiries can be directed to the corresponding author.

## Abbreviations

The following abbreviations are used in this manuscript:

TMP	Thermomechanical Processing
BOR	Burgers Orientation Relationship s
KAM	Kernel Average Misorientation
FEM	Finite Element Method

## References

[1] Williams J.C., Boyer R.R. (2020). Opportunities and issues in the application of titanium alloys for aerospace components. Metals.

[2] Semiatin S.L. (2020). An overview of the thermomechanical processing of α/β titanium alloys: Current status and future research opportunities. Metall. Mater. Trans. A.

[3] Qiu J., Ma Y., Lei J., Liu Y., Huang A., Rugg D., Yang R. (2014). A comparative study on dwell fatigue of Ti-6Al-2Sn-4Zr-x Mo (x = 2 to 6) alloys on a microstructure-normalized basis. Metall. Mater. Trans. A.

[4] Bantounas I., Lindley T.C., Rugg D., Dye D. (2007). Effect of microtexture on fatigue cracking in Ti–6Al–4V. Acta Mater..

[5] Zhang K., Wu X., Davies C.H.J. (2017). Effect of microtexture on short crack propagation in two-phase titanium alloys. Inter. J. Fatigue.

[6] Briffod F., Shiraiwa T., Enoki M., Emura S. (2022). Effect of macrozones on fatigue crack initiation and propagation mechanisms in a forged Ti-6Al-4V alloy under fully-reversed condition. Materialia.

[7] Liu C., Xu X., Sun T., Thomas R., da Fonseca J.Q., Preuss M. (2023). Microstructural effects on fatigue crack initiation mechanisms in a near-alpha titanium alloy. Acta Mater..

[8] Zhao Z.B., Wang Q.J., Liu J.R., Yang R. (2017). Characterizations of microstructure and crystallographic orientation in a near-α titanium alloy billet. J. Alloys Compd..

[9] Zheng G., Tang B., Zhou Q., Mao X., Dang R. (2020). Development of a flow localization band and texture in a forged near-α titanium alloy. Metals.

[10] Germain L., Gey N., Humbert M., Bocher P., Jahazi M. (2005). Analysis of sharp microtexture heterogeneities in a bimodal IMI 834 billet. Acta Mater..

[11] Gey N., Bocher P., Uta E., Germain L., Humbert M. (2012). Texture and microtexture variations in a near-α titanium forged disk of bimodal microstructure. Acta Mater..

[12] Kodli B.K., Saxena K.K., Dey S.R., Pancholi V., Bhattacharjee A. (2015). Texture studies of hot compressed near alpha titanium alloy (IMI 834) at 1000 C with different strain rates. IOP Conf. Ser. Mater. Sci. Eng..

[13] Germain L., Gey N., Humbert M., Vo P., Jahazi M., Bocher P. (2008). Texture heterogeneities induced by subtransus processing of near α titanium alloys. Acta Mater..

[14] Sun T., Deng Y., Liu W., Teng H., Wang R., Sun C., Hao D., Zhou J. (2024). Substructure and texture evolution of a novel near-α titanium alloy with bimodal microstructure during hot compression in α + β phase region. J. Alloys Compd..

[15] Zheng G., Mao X., Tang B., Zhang Y. (2020). Evolution of microstructure and texture of a near α titanium alloy during forging bar into disk. J. Alloys Compd..

[16] Wang X., Wang M., Ye P., Fu W., Cao G., Han Q., Liu X., Wu H., Fan G. (2025). The effect of strain rate on macrozones of the α+ β phase region during thermal deformation in near-α titanium alloys. J. Mater. Res. Tech..

[17] Sun Z.C., Zhang J., Yang H., Wu H. (2015). Effect of workpiece size on microstructure evolution of different regions for TA15 Ti-alloy isothermal near-β forging by local loading. J. Mater. Process Technol..

[18] Xu S., Zhang H., Xiao N., Qiu R., Cui Z., Fu M. (2023). Mechanisms of macrozone elimination and grain refinement of near α Ti alloy via the spheroidization of the Widmannstätten structure. Acta Mater..

[19] Huang L., Sun Z., Cao J., Yin Z. (2021). The formation and evolution of macrozone in Ti-6242S alloy during thermo-mechanical processing. J. Alloys Compd..

